# Optical coherence tomography (OCT) angiography findings in retinal arterial macroaneurysms

**DOI:** 10.1186/s12886-016-0293-2

**Published:** 2016-07-22

**Authors:** Maged Alnawaiseh, Friederike Schubert, Pieter Nelis, Gabriele Wirths, André Rosentreter, Nicole Eter

**Affiliations:** Department of Ophthalmology, University of Muenster Medical Center, Albert-Schweitzer-Campus 1, Building D15, 48149 Muenster, Germany; Department of Ophthalmology, University of Würzburg, Würzburg, Germany

**Keywords:** OCT angiography, Retinal arterial macroaneurysms, Fluorescein angiography

## Abstract

**Background:**

Optical coherence tomography angiography is a novel imaging technique that allows dyeless in vivo visualization of the retinal and choroidal vasculature. The purpose of this study was to describe optical coherence tomography (OCT) angiography findings in patients with retinal arterial macroaneurysms (RAMs).

**Methods:**

Three eyes of three patients with RAMs were retrospectively included. Fundus photography, OCT, fluorescein angiography (FA), and OCT angiography were performed. The entire imaging data was analyzed in detail.

**Results:**

OCT angiography could detect the RAMs noninvasively without dye injection. By simultaneously observing the OCT scans, it was possible to determine the depth of the RAMs in the retina, to detect the exact localization in relation to the main vessel, and to determine the level of blood flow in the RAMs.

**Conclusions:**

OCT angiography can clearly visualize RAMs without use of a dye. It also allows layer-specific observation of blood flow in each layer of the RAM. OCT angiography provides additional dynamic information on RAMs, which is not obtained with FA and facilitates a better understanding of its morphology and activity. This information in combination with ICG and fluorescein angiography can help to optimize direct laser treatment.

## Background

Arterial retinal macroaneurysms (RAMs) represent an acquired vascular disorder. They occur most frequently in elderly women suffering from arterial hypertension and/or atherosclerotic changes [1]. The macroaneurysms are usually round dilations of the large arterioles of the retina within the first three branches of the central retinal artery [1]. In the natural course of the pathology, there is a gradual and spontaneous regression in most cases with a good visual prognosis. However, retinal hemorrhage, vitreous hemorrhage or macular edema may occur as complications and lead to a decrease in visual acuity [1, 2]. Therapy is only indicated in patients with impaired visual acuity. Laser photocoagulation can be performed directly on the retinal macroaneurysm or indirectly by surrounding the macroaneurysm with laser points. There is no consensus as to whether direct or indirect treatment yields better results [3]. Intravitreal anti-vascular endothelial growth factor therapy appears promising as an alternative to laser treatment in cases of retinal macroaneurysms with macular exsudation [4, 5].

At present, different imaging modalities are used in the diagnosis of RAMs [6–8]. The imaging methods fluorescein angiography (FA) and spectral-domain optical coherence tomography (OCT) are widely used for diagnosis and follow-up of patients with RAMs [6, 7]. Saccular or fusiform dilation of the arteriolar wall is a pathognomonic sign. Angiography is particularly important for diagnosis when hemorrhaging obscures the vasculature. Late fluorescein leakage from within the areas of hemorrhage is characteristic of macroaneurysms and may assist diagnosis when the vasculature is not visible on direct examination [1, 2, 6].

Recently, a new noninvasive imaging technique has been developed using split-spectrum amplitude-decorrelation angiography software. OCT angiography enables a noninvasive dyeless visualization of blood flow in normal and pathologic vascularization in different retinal layers [9–11]. Ours is the first study to present OCT angiographic findings in RAMs.

## Methods

We retrospectively evaluated 3 eyes of 3 patients with RAMs. All patients underwent a complete ophthalmic examination including best-corrected visual acuity (BCVA), anterior segment examination, intraocular pressure measurement, dilated fundus biomicroscopy, color fundus photography, fluorescein angiography and spectral-domain OCT (Heidelberg Spectralis, Heidelberg, Germany). Additionally, all patients underwent OCT angiography using the split-spectrum amplitude-decorrelation angiography (SSADA) algorithm (RTVue XR Avanti with AngioVue; Optovue Inc, Fremont, California, USA). The new technology has been described in detail in previous publications [9–12]. The study was approved by the Ethics Committee of the University of Muenster, North Rhine Westphalia, Germany and was performed according to the tenets of the Declaration of Helsinki.

“Color-coding:

Color-coded, composite en-face OCT-angiograms were created to simplify comparison to the familiar ICG and fluorescein angiography, the OCT angiograms of individual layers were stained using Adobe Photoshop™. The colors assigned to the individual layers were red (superficial vascular plexus), white (outer retina), blue (deep retina) and green (choriocapillaris). Composite images were created by merging the images of all four layers with Adobe Photoshop™.”

## Results

Three eyes of three patients (3 women) were included, the patients ranging in age from 68 to 74 years, with a mean age of 71.3 ± 2.5 years. All patients had a documented history of arterial hypertension. OCT angiography images of acceptable quality were acquired in all cases.

### Case 1

**Fig. 1:**
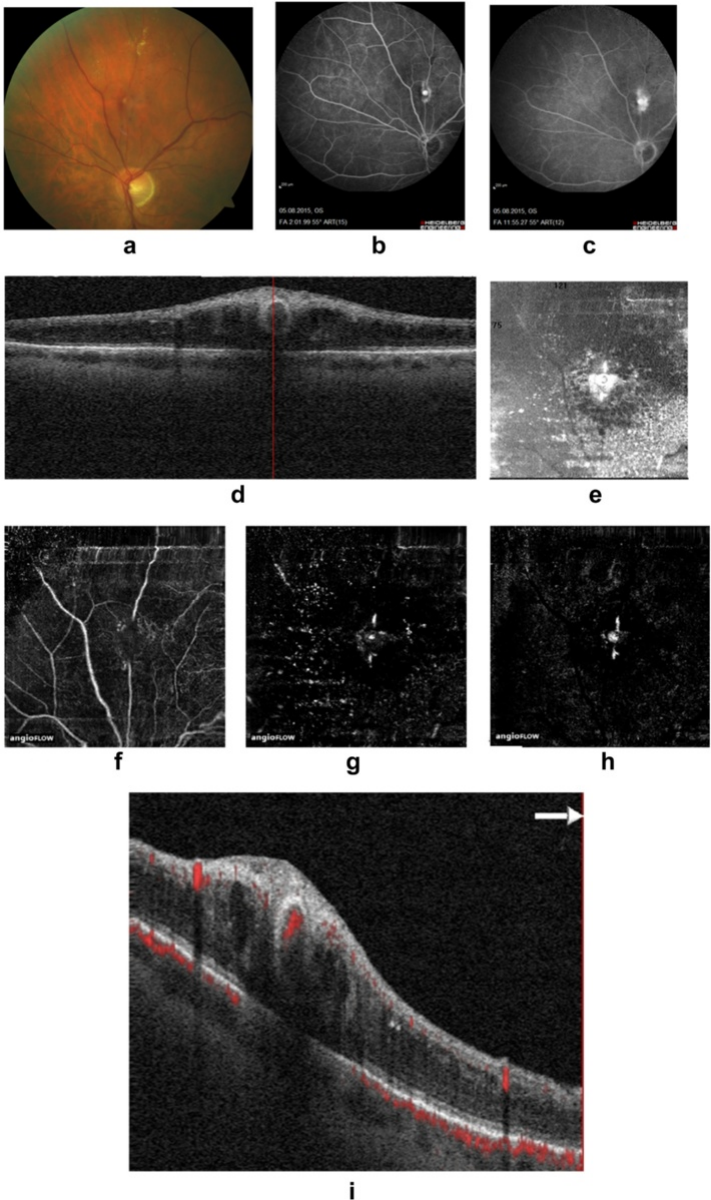
Fundus photography image **a** and early **b** and late **c** fluorescein angiogram showing a RAM superior to the optic disc. **d** Cross-sectional OCT. Sectional and **e** en face OCT image en face optical coherence tomography (OCT) images of the RAM. OCT angiograms of the different retinal layers showing an interruption of blood flow in the superficial OCT angiogram **f** and a significant flow in the deep OCT angiogram **g** and an OCT angiogram of the outer retina **h**. Cross-sectional OCT angiography visualizes blood flow in the RAMs **i**

A 74-year-old female noted visual impairment in her left eye. The best corrected visual acuity (BCVA) was 0.25 in the left eye. Fundus photography (Fig. 1a) shows retinal hemorrhage with a white lesion superior to the optic nerve. FA revealed staining in the early phase (Fig. 1b) and leakage in the late phase (Fig. 1c). The OCT showed a round cavity with a hyperreflective wall and a dark lumen in the area of the RAM (Fig. 1d). Cystoid retinal edema was adequately visualized in the en face OCT image (Fig. 1e).

The OCT angiogram also showed the RAMs. An interruption of blood flow in the area of the RAM was observed in the superficial retinal OCT angiogram (segmented with an inner boundary at 3 μm beneath the internal limiting membrane and outer boundary at 15 μm beneath the inner plexiform layer) (Fig. 1f). The deep retinal OCT angiogram (segmented with an inner boundary at 15 μm beneath the inner plexiform layer and the outer boundary at 70 μm beneath the inner plexiform layer) and the OCT angiogram of the outer retina showed high blood flow in the RAM (Fig. 1g and h). Using cross-sectional OCT angiography, it is possible to visualize the level of blood flow in the RAMs (Fig. 1i).

### Case 2

**Fig. 2:**
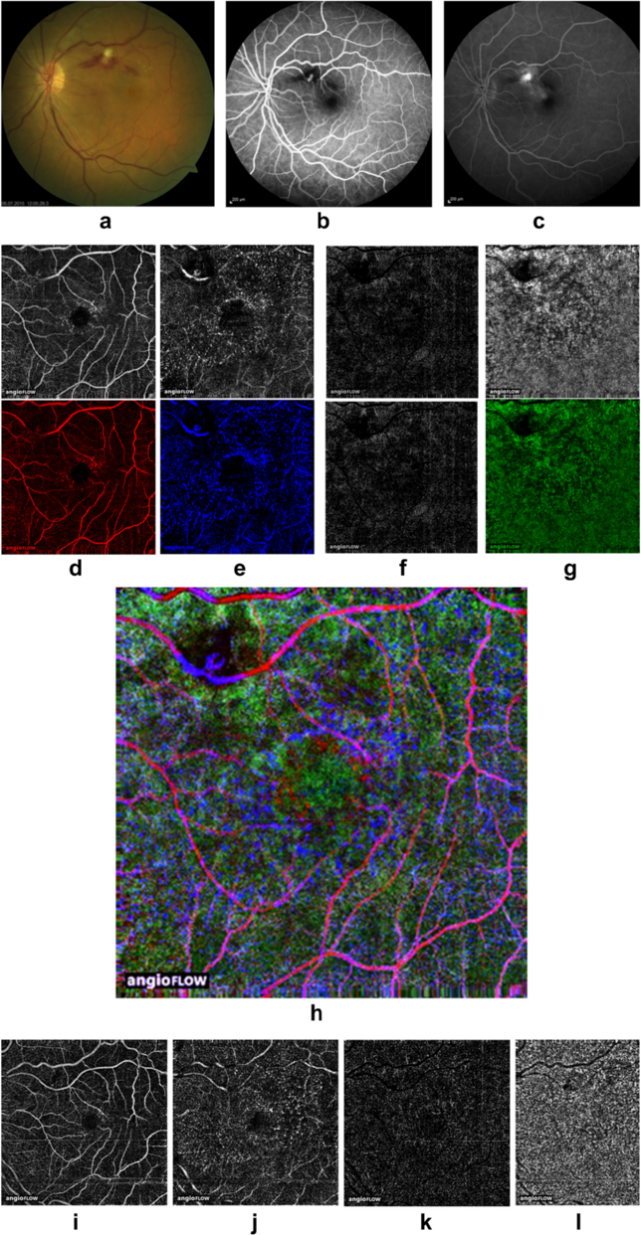
Fundus photography image **a** and early **b** and late **c** fluorescein angiogram showing a superotemporal RAM. OCT angiograms, color-coded OCT angiograms **d**-**g** and composite OCT angiograms **h** of the different retinal layers: the RAM is best visualized in the deep OCT angiogram. **i**-**l** OCT angiograms of the different retinal layers two months after intravitreal anti-vascular endothelial growth factor therapy

A 68-year-old female noted acute visual loss in her left eye, the BCVA was 0.1. Fundus photography, FA and OCT angiography findings of a superotemporal macroaneurysm with intraretinal hemorrhage are presented in Fig. 2a-h.

The superficial retinal OCT angiogram shows reduced blood flow in the RAM (Fig. 2d). Blood flow was detected in the same area in the deep retinal OCT angiogram (Fig. 2e) while no flow was detected in the outer retinal OCT angiogram (Fig. 2f). In this case, the RAM must have its origin in the inferior part of the vessel, as the saccular RAM can be perfectly visualized in the deep retinal OCT angiogram (Fig. 2e). Two months after intravitreal anti-vascular endothelial growth factor therapy it was not possible to visualize the RAM despite varying the segmentation (Fig. 2i - l).

### Case 3

**Fig. 3:**
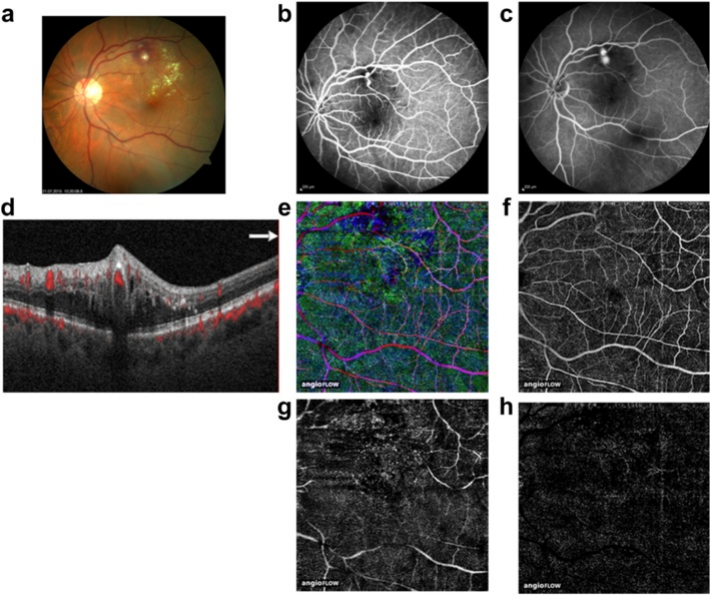
Fundus photography image **a** early **b** and late **c** fluorescein angiogram showing the two main parts of a superotemporal RAM. OCT angiograms **g**-**i** and composite OCT angiograms of the RAM showing blood flow in the superficial **f** and deep OCT angiograms **g**. **d** Cross-sectional OCT angiography

A 72-year-old female developed a deterioration of visual acuity in the left eye. The RAM is superotemporal in relation to the fovea, as in Case 2 (Fig. 3a). Using information from FA, SD OCT and OCT angiography (Fig. 3b-h), two main parts can be determined in the RAM: a smaller part (Fig. 3b) located mainly above the retinal vessel with blood flow seen in the superficial retinal OCT angiogram (Fig. 3f) and a fusiform part located below the retinal vessel (Fig. 3b) with blood flow seen mainly in the deep retinal OCT angiogram (Fig. 3g). The shape of the RAM is shown best on the early fluorescein angiogram (Fig. 3b). The cross-sectional OCT angiography visualizes the level of blood flow in the RAMs (Fig. 3d).

## Discussion

Fluorescein angiography is currently the standard method for diagnosis of vascular diseases of the retina. With the introduction of OCT angiography, a noninvasive diagnostic technique became available, which could reduce the range of indications of classic invasive fluorescein angiography. Dye injections can cause adverse reactions such as nausea or, rarely but critically, anaphylaxis, even in healthy subjects [13]. Optical coherence angiography was first reported using Doppler OCT [14]. Following this development, various three-dimensional (3-D) vascular imaging procedures based on OCT technology were introduced [15, 16]. Using newly developed OCT angiography, visualization of the inner and outer retinal vascular plexi and the choriocapillary layer is now possible without dye injection. This novel technology has been described as a useful tool in the diagnosis or follow-up of various retinal or choroidal vascular pathologies, and has been used in diabetic retinopathy, chronic central serous chorioretinopathy and age-related macular degeneration [9–11]. In the present study we demonstrate OCT angiography findings in patients with RAMs.

Up to now, the most reliable and common methods for detection of RAMs have been ICG and fluorescein angiography (FA) [1, 17]. However, we achieved noninvasive detection of RAMs without dye injection in all our cases by means of SSADA based OCT angiography. Furthermore, two features make OCT angiography preferable to classic FA: a precise three-dimensional (3D) localization of the RAM and localization of the blood flow in different retinal layers. Moreover, OCT angiography allows the examiner to vary the segmentation and to scroll through the different retinal layers in order to optimise the 3D localization of the RAMs.

Miura et al. demonstrated the clinical utility of Doppler OCT to evaluate RAMs [8]. In this paper Miura et al. reported, that Doppler OCT could detect only some parts of the retinal vasculature compared with ICG and fluorescein angiography. In one case the RAM was located in the medium layer of the retina with the presence of inner retinal hemorrhage, impeding the detection of RAMs by Doppler OCT imaging [8]. Taking this into account, it is important to mention that in this paper using Doppler OCT no layer-specific en-face OCT angiograms were presented. However the comparison between the different modalities of OCT angiography is a very interesting aspect, which has to be evaluated in further studies.

By simultaneously analyzing the information provided by standard SD OCT (retinal layer, en face image and thickness map) and OCT angiography, which are brought together by the software in the same computer-frame, it was possible to evaluate the exact topographic location of the RAM in three dimensions and to differentiate between thrombosed volume and volume with blood flow. This information is necessary for long-term follow-up and evaluation of treatment options such as laser coagulation.

The OCT angiography technique has a number of limitations: images are restricted to a small area, the objective is fixed and inflexible, and patients have to fixate precisely for several seconds, making imaging in the retinal periphery difficult to perform. Furthermore, with OCT angiography it is not possible to visualize and evaluate the breakdown of the blood-retinal barrier, which is usually represented by fluorescein leakage in FA [10, 11]. One of the important limitations of OCT angiography relating to RAMs is that changes in vessels or RAM visualized on OCT angiograms do not directly indicate the structural and morphological constitution of the RAM, because the OCT angiograms depict only blood flow. This must be taken into consideration, especially in cases with partial thrombosed RAM. However, despite these limitations, we consider OCT angiography a useful noninvasive method for visualization of important details of the retinal RAM without injection of dye.

Our study is limited by the small sample size. Further studies in larger groups of patients with a focus on the analysis of long-term findings would therefore be helpful to assess the definitive role of this new imaging tool in the diagnosis and follow-up of RAMs.

## Conclusions

This study presents OCT angiography findings in patients with RAM. With this new imaging technique we were able to visualize the level of blood flow in the RAMs. Simultaneous observations with standard OCT allowed exact localization of the RAM in the different retinal layers and assessment of levels of blood flow. OCT angiography provides additional dynamic information about the RAMs, which is lacking on FA. It can therefore be seen as an alternative, noninvasive tool for evaluation of RAMs, which enables a better understanding of their morphology and activity.

## Abbreviations

3D, three dimensional; BCVA, best-corrected visual acuity; FA, fluorescein angiography; OCT, optical coherence tomography; RAM, retinal arterial macroaneurysms; SSADA, split-spectrum amplitude-decorrelation angiography
